# Tranexamic acid: a narrative review of its current role in perioperative medicine and acute medical bleeding

**DOI:** 10.3389/fmed.2024.1416998

**Published:** 2024-08-07

**Authors:** Marwan Bouras, Alexandre Bourdiol, Paul Rooze, Yannick Hourmant, Anaïs Caillard, Antoine Roquilly

**Affiliations:** ^1^CHU Brest, Anesthesiology and Intensive Care Unit, Brest, France; ^2^INSERM UMR 1064 CR2TI, University of Nantes, Nantes, France; ^3^CHU Nantes, Anesthesiology and Intensive Care Unit, CIC Immunology and Infection, Nantes, France

**Keywords:** tranexamic acid, fibrinolysis, hemorrhage, trauma, postpartum hemorrhage, scheduled surgery, acute care

## Abstract

**Purpose:**

Tranexamic acid (TXA) is the most widely prescribed antifibrinolytic for active bleeding or to prevent surgical bleeding. Despite numerous large multi-center randomized trials involving thousands of patients being conducted, TXA remains underutilized in indications where it has demonstrated efficacy and a lack of harmful effects. This narrative review aims to provide basic concepts about fibrinolysis and TXA’s mode of action and is focused on the most recent and important trials evaluating this drug in different hemorrhagic situations.

**Methods:**

We selected every low bias RCT, and we highlighted their strengths and limitations throughout this review.

**Principal findings:**

While TXA appears to have a favorable benefit–risk ratio in most situations (trauma, obstetrics, at-risk for bleeding surgeries) evidence of benefit is lacking in certain medical settings (SAH, digestive bleeding).

**Conclusion:**

Although in some situations the drug’s effect on significant outcomes is modest, its favorable safety profile allows it to be recommended for trauma patients, in obstetrics, and in scheduled surgeries at risk of bleeding. However, it cannot be recommended in cases of spontaneous intracranial bleeding, subarachnoid hemorrhage (SAH), or gastrointestinal bleeding.

## Introduction

Tranexamic acid (TXA) is the most commonly prescribed antifibrinolytic for the management of active hemorrhage or to prevent bleeding in hemorrhagic surgery (1). TXA is recommended in guidelines worldwide and is classified as an essential medicine by the World Health Organization (WHO) (2). Despite many large multi-center randomized trials with thousands of patients being performed, TXA remains underutilized in indications where it has demonstrated efficacy and a lack of harmful effects. While TXA has been successfully employed to prevent or decrease blood loss in a variety of clinical conditions characterized by excessive bleeding (3), this drug is still the subject of large-scale trials in recent years. The aim of this narrative review is to provide a summary of the literature on TXA in order to give healthcare professionals all the information they need about its use. While TXA has been studied in numerous small retrospective and prospective studies, we focused on the largest and most recent trials with the greatest therapeutic impact.

## Methods

We undertook a targeted literature review including randomized controlled trials, guidelines and meta-analyses published in English to provide the readers the highest quality data. The literature review was performed for studies relating to the TXA use in adult patients suffering of acute hemorrhage or in prevention of surgical bleeding. Keywords used were: “tranexamic acid,” “trauma,” “traumatic brain injury,” “hemorrhagic shock,” “hemorrhage,” “post-partum hemorrhage,” “obstetric,” “anti-fibrinolytic therapy,” “scheduled surgery,” “cardiac surgery,” “orthopedic surgery,” “urologic surgery,” “subarachnoid hemorrhage,” “intracerebral hemorrhage” and “gastro-intestinal bleeding.” We selected every low bias RCT and we highlighted their strengths and limitations throughout this review.

## Fibrinolysis

**Figure 1:**
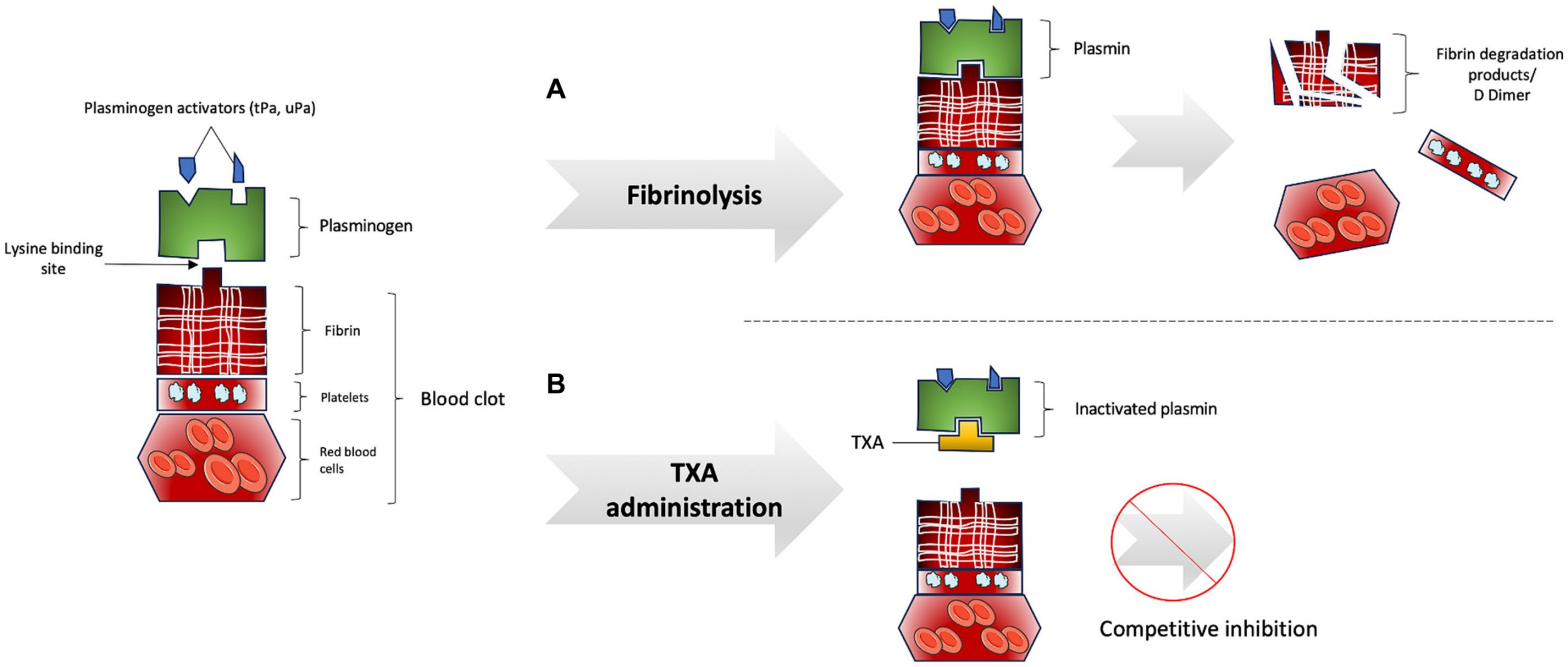
**(A)** Fibrinolysis process. **(B)** Inhibition of fibrinolysis process by tranexamic acid administration.

Fibrinolysis is a physiological process aimed at dissolving blood clots, thereby preventing vascular occlusion. Immediately following vessel injury, blood clot formation serves as the primary mechanism to prevent hemorrhage. A blood clot is composed of platelets (forming the primary hemostatic plug), red blood cells, and fibrin, a fibrillary protein that provides structural and mechanical stability to the clot. Coagulation is effective when thrombin converts fibrinogen into fibrin. Once the clot is formed and hemorrhage is halted, the process of fibrinolysis commences (4). The central enzyme in fibrinolysis is plasmin, which is the activated form of plasminogen. The activation of plasmin from plasminogen is regulated by activator proteases, specifically tissue plasminogen activator (tPA) and urokinase plasminogen activator (uPA). Plasminogen and its activating proteases bind to fibrin via the “lysine binding site,” leading to plasmin activation and the subsequent breakdown of the blood clot into fibrin degradation products (FDP) and D-dimer (5) (Figure 1). Activated plasmin also contributes to the degradation of the basement membrane and extracellular matrix, exhibiting pro-inflammatory activity through chemoattraction and complement activation to promote the healing of injured tissue.

Plasmin is physiologically modulated by specific inhibitors such as α2-antiplasmin and α2-macroglobulin, while plasminogen activators are regulated by plasminogen activator inhibitor-1 and -2 (PAI-1 and PAI-2) (6). Hemostasis necessitates a balance between bleeding and thrombosis, which is maintained by the activity of activators and inhibitors of fibrinolysis.

In certain clinical situations, such as trauma, postpartum hemorrhage, or surgery, the inhibitory mechanisms of fibrinolysis may fail, leading to excessive activation and potentially massive bleeding, a condition known as hyperfibrinolysis. Hyperfibrinolysis remains a major cause of death following multiple traumas (7, 8). Conversely, thrombosis occurs when the levels of lysis inhibitors surpass those of activators, resulting in reduced fibrinolysis and persistent clot formation (9).

## Tranexamic acid

The story of this small drug began in Japan in the 1960s. Utako Okamoto, a professor of physiology at the University of Keio, recognized that postpartum hemorrhage (PPH) was a leading cause of maternal mortality. She began her research with epsilon-amino-caproic acid (EACA) and subsequently developed tranexamic acid (TXA), which is 27 times more potent than EACA (10). However, Okamoto was hindered from conducting therapeutic trials of TXA in PPH due to the obstetricians’ rejection of this therapy. She passed away in 2016 before the results of the largest randomized trial of TXA in obstetrics were published (11).

TXA is a synthetic lysine-analog antifibrinolytic that competitively and reversibly inhibits the activation of plasminogen to plasmin (12). To inhibit fibrinolysis, TXA binds to plasmin via its “lysine-binding site,” preventing the coupling of fibrin and plasminogen and thereby inhibiting plasmin activation and fibrin degradation (Figure 1B). TXA has a half-life of approximately 2 h and is minimally metabolized in the liver, with 95% of the drug being excreted unchanged by the kidneys and urinary tract. Urinary excretion of TXA decreases with increasing plasma creatinine levels, necessitating dosage adjustments in patients with renal impairment and avoiding administration in patients with severe renal impairment (13).

TXA is well-tolerated, with mild adverse effects such as nausea, vomiting, and visual disturbances. Common contraindications include recent thrombosis (within the past 3 months) and seizures, as TXA is a competitive antagonist for GABAA and glycine receptors, which are inhibitory receptors in the central nervous system, thereby inducing hyperexcitability and seizures in a dose-dependent manner (14).

## The safety profile of TXA

The primary concerns regarding the use of TXA are based on these two main side effects: vascular-occlusive events and seizures.

### Vascular-occlusive events

Vascular-occlusive events (VOE) are exhaustively reported in the large RCTs covered in this narrative review. In the most recent trials, TXA has not been associated with VOE in trauma, traumatic brain injury (TBI), obstetrics, or scheduled surgery. Importantly, these trials encompass largely heterogeneous populations, with the incidence of VOE starting at 0.3% in obstetrics, increasing to 1.7% in both the CRASH-2 (15) and CRASH-3 (16) trials, and peaking at 13.9% in the PATCH trial (17). The higher incidence in the PATCH trial is attributed to its inclusion of only patients at risk of coagulopathy and more frequent screening for deep-vein thrombosis using Doppler.

Even when considering a composite cardiovascular outcome, including myocardial injury after non-cardiac surgery (MINS), no difference has been found between patients treated with TXA or placebo (18), despite the high incidence of the composite endpoint.

To the best of our knowledge, the only large-scale RCT in which TXA was found to be harmful is the HALT-IT trial, where VOE were more frequent in the intervention arm (0.8% vs. 0.4%, RR 1.84, CI 1.15 to 2.98), although the total incidence of arterial thromboembolic events (myocardial infarction or stroke) did not differ significantly. It is noteworthy that the TXA dose administered used in HALT-IT was slightly higher than in obstetrics, trauma, or non-cardiac surgery, with a loading dose of 1 g followed by a maintenance dose of 3 g over 8 h.

### Seizures

If VOE appears to be a natural concern when considering the adverse effects of an antifibrinolytic drug, seizures are not. The first warning emerged from cardiac surgery theaters, where the drug has been extensively used for the past 20 years (19), soon followed by other reports in patients with no previous history of seizures (20).

These observations prompted intense research into the mechanisms underlying TXA-associated seizures. Neuronal hyperexcitability due to TXA could be mediated through both γ-Aminobutyric acid type A (GABAA) (21) and glycine receptors (14), of which TXA is a competitive antagonist. Both GABAA and glycine receptors are well-known for their role in neuroinhibition in physiological settings. Drugs affecting these receptors, such as sevoflurane, desflurane, or propofol, may dampen the effects related to TXA, explaining why TXA-associated seizures appear more frequently in the postoperative room and are less frequently reported in TBI or trauma trials.

TXA effects in *in vitro* studies on these receptors appear to be dose-dependent, consistent with the incidences of adverse events reported in clinical trials. When seizures were first observed, high-dose boluses up to 100 mg/kg were the norm in cardiac surgery, as evidenced by the initial protocol of the study led by Myles et al., focusing on patients undergoing coronary bypass surgery (22). This protocol was later modified to 50 mg/kg (after enrolling about one-third of the patients) due to the high incidence of seizures. In the final report, 15 patients (0.7%) experienced seizures in the intervention arm vs. 2 (0.1%) in the control group (RR 7.62, CI 1.77–68.71). These numbers are consistent with those later reported in the OPTIMAL trial (23), comparing high-dose TXA (bolus 30 mg/kg followed by a maintenance dose of 16 mg/kg/h) versus low-dose TXA (bolus 10 mg/kg followed by a maintenance dose of 2 mg/kg/h) in patients undergoing cardiac surgery. Indeed, the former experienced an incidence of seizures of 0.7% and the latter 0.4%. In non-cardiac surgery, the HALT-IT trial also found an association between TXA and postoperative seizures (0.6% vs. 0.4%, RR 1.73; 95% CI 1.03 to 2.93) with a maintenance regimen higher than those reported in trauma, non-cardiac surgeries, or obstetrics. Aside from the consistent dose-dependent effect observed in both *in vitro* and clinical studies, the specific toxicity in cardiac surgery may be explained by the disruption of the blood–brain barrier (24) and the intense neuroinflammation caused by extracorporeal circulation.

While these observations support the overall safety of TXA with respect to VTE, this does not apply to seizures, especially in high-risk patients undergoing cardiac surgery, where reasonable doses should be used.

## Therapeutic uses

### In trauma patients

About 40% of deaths in trauma patients are related to hemorrhagic shock (25). Hyperfibrinolysis contributes to coagulopathy and has an estimated incidence of 15% (26). In 2011, TXA suddenly garnered significant attention in traumatology with the CRASH-2 trial, a large, randomized, double-blind, placebo-controlled trial assessing the efficacy of TXA, which involved 20,211 adult trauma patients (15). Trauma patients with or at risk for significant hemorrhage were treated with intravenous TXA (1 g in 10 min and 1 g infused over 8 h) or placebo. TXA significantly reduced all-cause mortality at 28 days (RR = 0.91; 95% CI 0.85–0.97; *p* = 0.0035). Death due to bleeding was also reduced (4.9% vs. 5.7%, *p* = 0.007). There was no difference in vascular occlusive events (1.7% vs. 2.0%). In the years following this publication, TXA gained significant popularity, leading to its inclusion in the 2011 WHO’s list of essential medicines (2).

Subsequently, criticism of the CRASH-2 trial emerged: there were no differences in transfusion rates between the two groups, implying that the reduction in bleeding mortality by TXA was not solely due to its anti-fibrinolytic effect. Patients in the CRASH-2 trial were mainly treated in low-income countries where the availability of emergency surgery or advanced trauma care was low; would the same results be achieved in countries with high-performance trauma centers with better access to transfusion or embolization? The researchers did not use a protocolized method to identify vascular occlusive events, suggesting that these may have been under-reported, particularly in low-income countries where Doppler availability is inconsistent. An exploratory analysis of the CRASH-2 trial found that TXA was associated with a decreased risk of death due to bleeding when administered within 3 h of injury (27), with a concluding sentence that dampened the growing enthusiasm for the molecule: “for trauma patients admitted late after injury, tranexamic acid is less effective and could be harmful.”

As the reproducibility of trial results is the basis of scientific doctrine, two large-scale trials were carried out to confirm CRASH-2’s results. The STAAMP trial compared prehospital TXA 1 g to placebo in 903 trauma patients at risk of hemorrhage (28). TXA was not associated with a reduction in 30-day mortality (8.1% in TXA and 9.9% in placebo; HR = 0.81; 95% CI 0.59–1.11; *p* = 0.018), but 30-day mortality was lower when TXA was administered within 1 h of injury (4.6% vs. 7.6%, *p* < 0.002). Patients with severe shock who received TXA demonstrated lower 30-day mortality (18.5% vs. 35.5%, *p* < 0.003). Adverse events were similar between groups. A secondary analysis of the STAAMP trial showed that the administration of TXA within 1 h of injury in patients at risk of hemorrhage was associated with a 30-day survival benefit, lower incidence of multiple organ failure, and lower transfusion requirements (29).

The second trial aiming to confirm the results of the CRASH-2 trial is the PATCH trial (Pre-hospital Antifibrinolytics for Traumatic Coagulopathy and Hemorrhage) (17). It included 1,310 adult trauma patients at risk of trauma-induced coagulopathy [based on the COAST score, which identified a group of patients with acute traumatic coagulopathy using prehospital observations like prehospital SBP, prehospital thorax drain, etc. (30, 31)] and in whom TXA could be given within 3 h after trauma. There was no difference in the primary outcome (favorable Extended Glasgow Outcome Score at 6 months), 53.7% in the TXA group and 53.5% in the placebo group. At 28 days after injury, 17.3% in the TXA group and 21.8% in the placebo group had died (RR = 0.79; 95% CI 0.6–0.99). By 6 months, 19% in the TXA group and 22.9% in the placebo group had died (RR = 0.83; 95% CI 0.67–1.03). Serious adverse events, including vascular occlusive events, did not differ statistically between the groups. Here, the primary endpoint was Glasgow Outcome Scale-Extended (GOS-E) at 6 months, a questionable outcome for an emergency treatment administered in the prehospital setting. Functional outcome was unchanged at 6 months, but trauma patients can continue to progress after 6 months, and good recovery is a subjective notion. Indeed, following traumatic brain injury (TBI), between one-third and one-half of patients with severe disability according to the GOS-E reported health-related quality of life within the normal range (32). The effectiveness of TXA in reducing deaths in a high-income country is the main result of this trial. It confirms the results of CRASH-2 and the safety profile of TXA in trauma patients. An antifibrinolytic treatment that consistently reduces mortality at 24 h and 28 days without adverse effects can be widely recommended (33).

In summary, TXA is recommended for severe trauma patients and may be provided in the prehospital setting within 3 h of trauma. TXA given beyond this point may be harmful and should be avoided.

### In traumatic brain injury

TBI is a leading cause of death and disability worldwide, contributing to 30% of trauma-related deaths (34). In the aftermath of the CRASH-2 trial, researchers investigated whether TXA could be beneficial for isolated TBI. Numerous small-scale studies were conducted, with some finding no effect of TXA (35, 36), and others showing a reduction in intracranial hemorrhage without clinical significance (37, 38). To fully investigate the efficacy of TXA in TBI, the CRASH consortium developed the CRASH-3 trial (16). In this trial, 9,202 adults with TBI and Glasgow Coma Scale <13 or intracranial hemorrhage on CT scan, excluding patients with major extracranial hemorrhage, were randomized within 3 h of TBI (this was changed during the trial from 8 h to 3 h). Head injury-related death was 18.5% in the TXA group versus 19.8% in the placebo group (RR = 0.94, 95% CI 0.86–1.02). A sensitivity analysis excluding the most severe patients (Glasgow score of 3 or bilateral unreactive pupils) showed the same absolute reduction in mortality without reaching statistical significance (12.5% with TXA and 14.0% with placebo; 95% CI = 0.8–1.0). In subgroup analyses, TXA reduced the risk of head injury-related death in patients with mild-to-moderate TBI (Glasgow score = 9–12) but not in patients with severe TBI (GCS 3–8) or in those whose pupils were not reactive. An effect on time to treatment was observed, but only in patients with mild to moderate TBI.

The publication of the CRASH-3 trial was widely criticized. Firstly, the primary endpoint was not the one mentioned in the clinical trial protocol (NCT01402882). Head injury-related death was thus highlighted in the publication. Although the use of disease-specific mortality as a meaningful endpoint may be used in trials lacking power to prove an effect on all-cause mortality, it does not seem appropriate for a study involving about 10,000 patients. Moreover, this outcome is subjective: it is difficult to categorize the cause of death of a patient suffering from intracranial hypertension requiring hypothermia and a barbiturate coma and dying of pneumonia potentiated by the introduction of these therapies. Other criticisms of this trial include the desire for it to be positive, even if this means highlighting the results of a secondary endpoint, and the need for a meta-analysis at the end of the article to reach statistical significance thresholds. However, once again, the risk of vascular occlusive events and seizures were similar in both groups.

The second large RCT evaluating TXA included 966 patients with moderate-to-severe TBI (GCS ≤12 and without hemorrhagic shock, systolic blood pressure ≥ 90 mmHg) (39). There was no difference in good functional outcome at 6 months (65% in TXA group vs. 62% in the placebo group, *p* = 0.84). All-cause mortality at day 28 was similar, although there was a trend toward a positive effect with TXA (14% vs. 17%; *p* = 0.26). Adverse events were similar between groups. A meta-analysis including nine randomized trials found that in patients with acute TBI, TXA probably has no effect on mortality or disability but may decrease hematoma expansion on subsequent imaging (40). The use of TXA did not increase the risk of adverse events.

In summary, trials are less conclusive on the use of TXA in isolated TBI. The treatment seems to be more effective in less severe patients. However, in the absence of treatment-related complications, it may be legitimate to use this treatment. The ongoing CRASH-4 trial will evaluate the effects of early intramuscular TXA on intracranial hemorrhage, disability, death, and dementia in elderly patients with symptomatic mild traumatic brain injury.

### In obstetric patients

#### Postpartum hemorrhage

The evaluation of TXA in postpartum hemorrhage (PPH) is closely linked to its history, as it was developed for this indication. The initial trials on this subject were small, randomized, open-label studies. One such trial examined high-dose TXA (4 grams over 1 h, then 1 gram per hour for 6 h) in 144 women with PPH who had lost more than 800 mL of blood. There was only a 48 mL difference in total blood loss 6 h after vaginal delivery between the groups, and the trial was not adequately powered to address safety issues (41).

The emblematic trial of TXA in PPH is the WOMAN trial (11), a large RCT including 20,060 women with PPH worldwide, randomized to receive either placebo or TXA. There was no difference in the composite primary endpoint of all-cause mortality or hysterectomy (5.3% vs. 5.5%, *p* = 0.65). Death from PPH was 1.5% in the TXA group versus 1.9% in the placebo group (RR = 0.81; 95% CI = 0.65–1.00; *p* = 0.045). A subgroup analysis of women treated with TXA within 3 h of giving birth demonstrated a significant reduction in death from PPH. Adverse event rates were also similar. In this trial, as in the CRASH-3 trial, emphasis was placed on secondary disease-specific mortality outcomes, relegating the results of the primary outcome to second place. This has led part of the scientific community to disregard the results of this trial, even though it was a high-quality study from which conclusions can be drawn. The effect on mortality, albeit small, should be considered for a treatment with no adverse effects. Additionally, a meta-analysis of the CRASH-2 and WOMAN trials, published at the end of the WOMAN trial draft, estimated that each 15-min delay in TXA treatment results in a 10% reduction in survival following hemorrhage, and that no benefit is observed after 3 h. This must be taken into account when administering treatment.

#### Prevention of post-partum hemorrhage

The role of TXA in the prevention of PPH has also been evaluated in large, randomized trials. The TRAAP trial (42) randomized 3,891 women in labor who had a planned vaginal delivery to receive 1 g of TXA or placebo. There was no difference in the primary outcome of blood loss of at least 500 mL (8.1% vs. 9.8%, *p* = 0.07). There were a few secondary outcomes that were statistically significant in favor of TXA (e.g., less use of uterotonic agents), but there were no differences in total blood loss, blood transfusion needs, or requirements for surgery or embolization. The incidence of thromboembolic events in the 3 months after delivery did not differ, but the frequency of vomiting or nausea in the delivery room was higher in the TXA group (7.0% vs. 3.2%, *p* < 0.001), which can be a disabling side effect in this setting.

The same French team evaluated TXA for the prevention of PPH after cesarean delivery in the TRAAP-2 trial (43). The primary outcome (postpartum hemorrhage >1,000 mL or red-cell transfusion within 2 days after delivery) occurred in 26.7% in the TXA group and 31.6% in the placebo group (*p* = 0.003), but the difference in blood loss between the groups was clinically irrelevant.

In summary, as recommended by American (44), French (45), and European (46) guidelines, the early use of TXA for all cases of PPH should be part of the standard of care, even if the evidence supporting it appears weaker than in trauma patients. The use of TXA for PPH prevention in vaginal deliveries cannot be recommended. As for its use for PPH prevention in C-sections, it remains at the discretion of the physician.

### Two questions about TXA administration in hemorrhagic conditions

#### Timing of administration?

**Figure 2:**
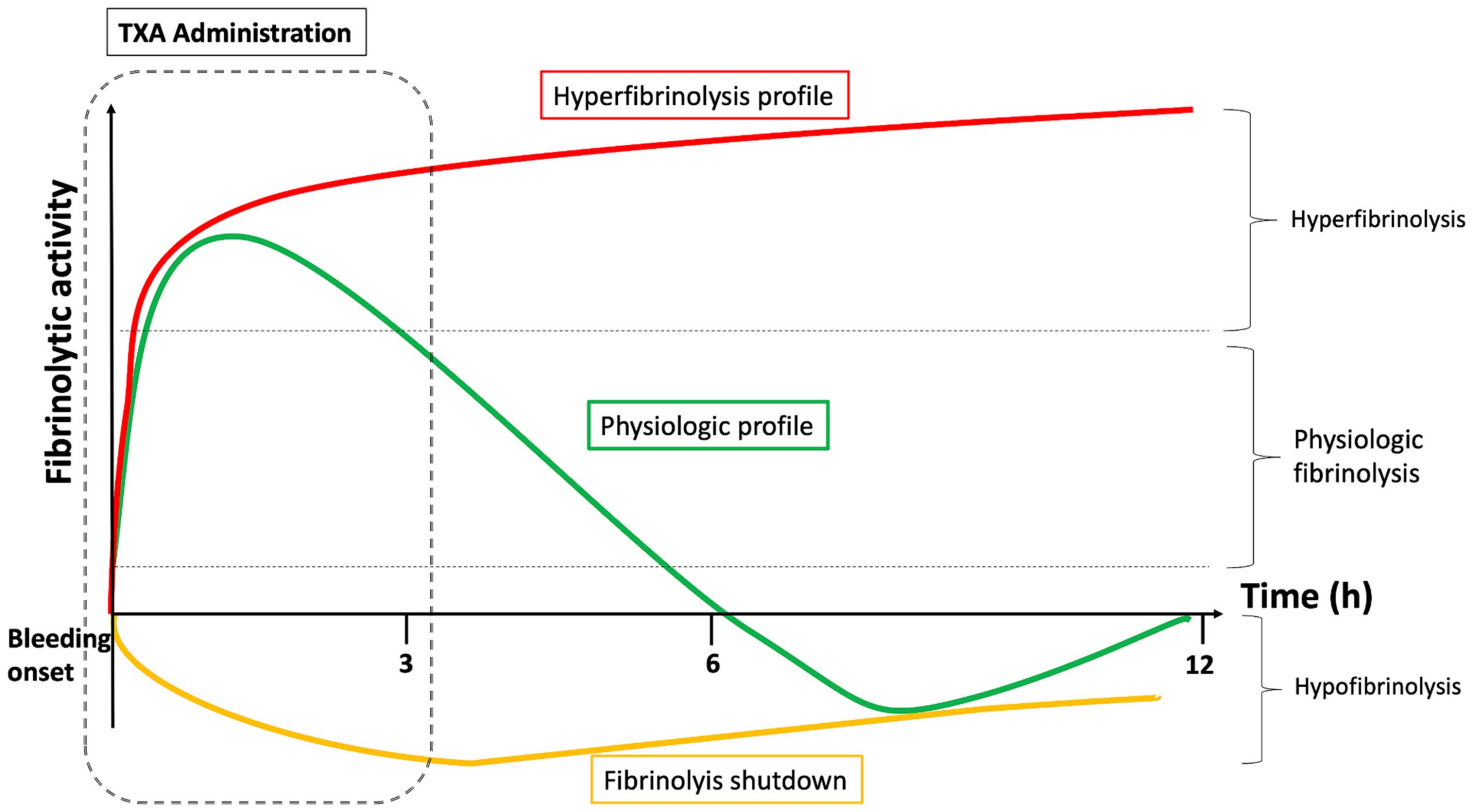
Different fibrinolysis profiles (physiologic fibrinolysis, hyperfibrinolysis and fibrinolysis shutdown) and effect of TXA administration timing.

After a trauma or childbirth, there are several fibrinolysis profiles: physiologic fibrinolysis, hyperfibrinolysis, and fibrinolysis shutdown (8) (Figure 2). Hyperfibrinolysis corresponds to an overactivation and is proposed as a pathological mechanism of trauma-induced coagulopathy. Conversely, the shutdown of fibrinolysis is a pathological process leading to a pro-coagulant state. In a cohort of 180 severely injured patients, the outcomes of these three profiles were analyzed (47). Mortality was lowest in the physiologic group (3%) compared to the shutdown (17%) and hyperfibrinolysis (44%) groups. Exsanguination represented 66% of deaths in the hyperfibrinolysis group, whereas the shutdown group experienced a higher mortality attributable to multiple organ failure (40% vs. 7%, *p* = 0.048).

TXA administration is intended for patients with hyperfibrinolysis as well as those with physiological fibrinolysis immediately after the onset of bleeding. Indeed, patients in the “physiological group” (i.e., most patients) develop physiological anti-fibrinolytic mechanisms, notably via alfa-1 antiplasmin. This adaptation takes place about 3 h after the trauma. This could explain the beneficial effect of TXA found in the CRASH-2 and WOMAN studies when administered within 3 h of trauma. The longer the delay between the injury and TXA administration, the greater the likelihood that the patient has low fibrinolytic activity. If antifibrinolytic treatment is administered more than 3 h after the trauma to these patients while they are in a state of natural anti-fibrinolysis, this exposes them to thrombotic complications or disseminated intravascular coagulation. For example, 65% of severely injured patients whose blood samples were taken 12 h post-injury had fibrinolysis shutdown (48), compared to less than 20% in the early phase (49).

Ideally, a viscoelastic test should be performed to individualize TXA administration, so that only hyper-fibrinolytic patients and “physiological” patients in the hyper-fibrinolytic phase are treated. However, this is impossible in the pre-hospital emergency setting. In this case, the benefit–risk ratio favors administering TXA within 3 h of the trauma, which will efficiently treat hyper-fibrinolytic patients (the most severe and those who are at risk of exsanguination) as well as physiological patients in the early phase. Fibrinolysis shutdown patients will be treated in excess, but they represent a small contingent of early-stage patients.

#### Anti-inflammatory effects?

One controversial aspects of TXA use is its mechanism of action. How could an antifibrinolytic drug reduce bleeding-related deaths in the CRASH-2 and WOMAN trials without reducing the need for transfusion? What protective effect could TXA have in TBI if it does not reduce the size of the hemorrhagic cerebral lesion? While some authors concluded that these data were sufficient to discount the effect of TXA, others looked for alternative explanations. Plasminogen and plasmin have inflammatory effects, including the activation of complement, the chemoattraction of leukocytes, neutrophils, monocytes, and macrophages, and the secretion of pro-inflammatory cytokines (50). Plasmin enhances the formation of the main complement proteins (C3 and C5) and activates complement fragments C3a/C5a (called anaphylatoxins), which recruit leukocytes and induce the membrane attack complex on macrophages (51), leading to an inflammatory state. Plasmin also acts as a proinflammatory mediator by triggering chemotaxis (52) and cytokine release (53).

Hemorrhagic shock is both hypovolemic and vasoplegic. Trauma-induced inflammation can be even more deleterious than blood loss, and hemodynamic failure correlates with the intensity of the systemic inflammatory response and is an independent factor in mortality (54). The beneficial effect of TXA could therefore be due to a restriction of the overwhelmed inflammatory response. TXA reduces C5a generation during tPA-mediated fibrinolysis by inhibiting plasmin and reducing post-traumatic inflammatory responses (55). In a rodent model of hemorrhagic shock, TXA suppressed the early increase of proinflammatory cytokine IL-1β and the later increase of anti-inflammatory cytokine IL-10 (56). Other effects of TXA beyond antifibrinolysis are described in the literature, such as the aforementioned anti-inflammatory activity or the stimulation of mitochondrial respiration and endothelial repair (57). This versatility, combined with the large scale achieved by past cohorts, should drive further studies to continue focusing on patient-related outcomes as primary endpoints.

### In medical causes of bleeding

#### Gastro-intestinal bleeding

The first large-scale randomized trials evaluating TXA were conducted in gastrointestinal (GI) bleeding. In 1983, Barer et al. demonstrated that TXA reduced mortality (6.3% vs. 13.5%, *p* = 0.0092) in 775 patients suffering from GI bleeding. The results were promising, but the RCTs had a high risk of bias, and the effect size was highly influenced by Barer’s RCT. In 2020, the HALT-IT trial (58) evaluated TXA in GI bleeding in over 12,000 patients. Patients received either a loading dose of 1 g TXA followed by a dose of 3 g TXA over 24 h, or a placebo. Death due to bleeding within 5 days (the primary outcome) occurred in 222 (4%) of 5,956 patients in the TXA group and in 226 (4%) of 5,981 patients in the placebo group. Rates of rebleeding, surgery, endoscopy, or the need for transfusion were also similar. However, deep vein thrombosis and pulmonary embolism were higher in the TXA group than in the placebo group (0.8% vs. 0.4%, *p* = 0.04). The reasons for the negativity of this trial may be due to the timing of the precise onset of GI bleeding being less clear than in the case of trauma or childbirth. The WOMAN and CRASH-2 trials showed that TXA was effective when administered within 3 h of injury. GI bleeding is an insidious disorder, and most patients in the HALT-IT trial were treated after the 8th hour. Additionally, GI bleeding occurs in a patient population wholly different from those with traumatic and postpartum hemorrhage. Those with GI bleeding were older (mean age 57 years in HALT-IT), and more than 70% of participants had significant comorbidities (including 41% with liver disease) known to be associated with an increased risk of VTE (59). The use of a higher regimen of TXA may also explain the increase in VTE.

In summary, the use of TXA in patients suffering from GI bleeding is not recommended and may even be harmful.

#### Spontaneous intracranial hemorrhage

##### Intracerebral hemorrhage

The TICH-2 trial (60) was the main randomized controlled trial comparing TXA with placebo in 2,325 patients with intracerebral hemorrhage (ICH). There was no difference in functional status at 90 days between the two groups. Despite a reduced, clinically irrelevant volume of hematoma (3.7 mL vs. 4.9 mL), mortality at 90 days was also similar. Most patients were enrolled after 3 h of symptom onset, which may contribute to the lack of effect of TXA. In a post-hoc analysis of the TICH-2 trial, TXA reduced the risk of early (OR = 0.79; 95% CI 0.63–0.99; *p* = 0.041) but not late neurological deterioration (61). Another exploratory analysis showed that TXA reduced the risk of ICH expansion in all patients. A small RCT of 100 patients with ICH does not provide evidence that TXA prevents intracerebral hemorrhage growth, although the treatment was safe with no increase in thromboembolic complications (62). Conversely, a meta-analysis showed that TXA could reduce hematoma expansion in ICH but had no notable impact on good functional outcomes or mortality (63).

##### Subarachnoid hemorrhage

The use of TXA in subarachnoid hemorrhage (SAH) is justified by the prevention of rebleeding. Interest in the use of TXA in SAH stems from an initial RCT of over 500 patients in 2002 (64), which found a reduction in the rebleeding rate from 10.8 to 2.4% and an 80% reduction in mortality related to early rebleeding. The favorable outcome according to the GOS increased from 70.5 to 74.8%. In 2013, a Cochrane meta-analysis (65) assessed that TXA did not decrease poor neurologic outcomes or mortality. There was a significant reduction in the rate of rebleeding (RR 0.64; 95% CI 0.44–0.97); however, it increased cerebral ischemia (RR 1.41; 95% CI 1.04–1.91). Conversely, in another recent meta-analysis of 4,883 patients which evaluated TXA in SAH and ICH, TXA was associated with reduced mortality (RR = 0.78; *p* = 0.002). However, most of the studies included were carried out before today’s rapid access to embolization, which remains the most effective treatment to prevent rebleeding in SAH. More recently, the ULTRA trial (66) investigated TXA’s effect on clinical outcomes in aneurysmal SAH to date. 955 patients with SAH were enrolled to receive TXA or placebo. TXA was given on average 3 h after symptom onset, and surgical management was done by an average of 14 h. Good clinical outcome (assessed by the modified Rankin Scale) was observed in 287 (60%) of 475 patients in the TXA group, and 300 (64%) of 470 patients in the control group (OR = 0.86; 95% CI 0.66–1.12). Rebleeding after randomization and before aneurysm treatment occurred in 49 (10%) patients in the TXA group and 66 (14%) patients in the control group (OR 0.71; 95% CI 0.48–1.04). A 2022 Cochrane meta-analysis does not support the routine use of antifibrinolytic drugs in the treatment of patients with aneurysmal SAH (67). Early administration with concomitant treatment strategies to prevent delayed cerebral ischemia did not improve clinical outcomes, and there was a trend toward delayed cerebral ischemia.

In summary, the most robust trials on the use of TXA in nontraumatic intracranial bleeding reported no benefit. This treatment may even increase delayed cerebral ischemia in the case of SAH.

### In scheduled surgery

Management of perioperative bleeding is complex and involves multiple assessment tools and strategies to ensure optimal patient care, with the goal of reducing morbidity and mortality. The use of antifibrinolytics such as TXA plays a central role in the management of intraoperative bleeding, reducing morbidity and mortality while having a strong socio-economic impact by limiting the need for transfusions—a precious resource—and reducing postoperative complications. Recent recommendations from the European Society of Anesthesia position TXA as a central component of perioperative bleeding management (68).

#### Non cardiac surgery

A growing number of adults undergo major non-cardiac surgery every year, including patients with more comorbidities and increased risks of bleeding and thrombotic events (69). A recent large RCT included 9,535 patients at increased cardiovascular risk who were scheduled to undergo non-cardiac surgery to receive 1 g TXA or placebo at the start and end of surgery (18). A composite bleeding outcome event (i.e., life-threatening bleeding, major bleeding, and bleeding into a critical organ) at 30 days after randomization occurred in 9.1% of the intervention group and in 11.7% of the placebo group (*p* < 0.001). Although they failed to demonstrate non-inferiority in a safety composite cardiovascular outcome event (14.2% in the TXA group vs. 13.9% in the placebo group), the statistical margin was stringent. The clinical implication of these results is that there is no excess risk of thrombotic events with the use of TXA to limit bleeding in scheduled non-cardiac surgery. This is consistent with a meta-analysis on 125,550 patients undergoing surgical procedures, in which total thromboembolic events were found in 2.1% of patients in the TXA group and 2% in the control group (70). Administration of TXA was associated with a significant reduction in overall mortality and bleeding mortality. The data from these studies alone recommend TXA for patients undergoing surgery at risk of bleeding (71), but there is a body of literature specific to each type of surgery:

##### Orthopedic surgery

The reduction of blood loss in orthopedic surgery is of great importance, especially in hip or knee arthroplasty. In a subgroup analysis of more than 2,000 patients undergoing orthopedic surgery in Devereaux’s study (18), TXA was more effective than placebo in reducing bleeding (HR = 0.72). Based on other trial results (72, 73), it is recommended to use prophylactic TXA to reduce blood loss and transfusion in patients with a significant risk for bleeding undergoing major orthopedic surgery such as total knee arthroplasty or total hip arthroplasty. There is also evidence for TXA reducing the need for blood transfusions during hip fracture surgery (74).

##### Gynecological surgery

In a double-blinded randomized placebo-controlled trial of 332 women undergoing laparoscopic or vaginal hysterectomy (75), both intraoperative total blood loss and the risk of reoperation were reduced in the group treated with TXA. Among women undergoing abdominal myomectomy, TXA appears effective in reducing perioperative blood loss compared to placebo (76). A review with a meta-analysis on different gynecological surgeries showed that during hysterectomy, TXA reduced blood loss, blood transfusion (12% vs. 42%, *p* < 0.00001), and decreased the risk of delayed hemorrhage in cervical conization (77).

##### Neurosurgical surgery

Patients undergoing intracranial neurosurgery were excluded from Devereaux’s study (18). A recent RCT in 30 patients undergoing meningioma resection showed a benefit of TXA by reducing perioperative blood loss by 46% and blood transfusion requirements (78). In a meta-analysis involving 200 patients operated on for brain tumors, TXA decreased blood loss, but the need for transfusion was not different between groups (79). For a long time, TXA was not indicated for neurosurgical patients because of concerns about seizures. None of the studies showed a significantly higher rate of convulsions when TXA was used in intracranial surgery (80–82).

A recent meta-analysis, including 23 studies (1,621 patients) (83), evaluated the efficacy of intravenous TXA in reducing perioperative blood loss and the need for transfusion during elective multilevel spine surgery. The findings demonstrated a significant reduction in perioperative blood loss with TXA administration (mean difference of 284.39 mL; 95% CI: 437.66 to 131.12 mL; *p* < 0.001), as well as a notable decrease in intraoperative blood transfusion requirements (mean difference of 333.78 mL; 95% CI: 540.45 to 127.01 mL; *p* = 0.002). No significant difference was found in the incidence and types of thrombotic complications when TXA was used in spinal surgery in another meta-analyses including 1,213 participants (RR =1.46, 95% CI: [0.65, 3.31], I^2^ = 0%, *p* = 0.36) (84).

#### Urologic and visceral surgery

TXA administration has been shown to decrease the need for blood transfusions during percutaneous nephrolithotomy (85). A meta-analysis of 9 studies found that TXA reduced intra-operative blood loss in prostate surgery without increasing the risk of deep vein thrombosis (DVT) or pulmonary embolism (86). Additionally, TXA decreased intraoperative blood loss during elective extrahepatic abdominal and pelvic surgery without increasing complications (87). The ongoing TRIGS trial (multicenter, pragmatic, double-blind, randomized clinical trial NCT04192435) will compare the incidence of surgical site infection and red cell transfusion requirements after intravenous tranexamic acid versus placebo in patients undergoing gastrointestinal surgery.

#### Cardiac surgery

Two randomized controlled trials evaluating TXA in cardiac surgery provide strong evidence to recommend its use in this setting. Among 4,631 patients undergoing coronary artery surgery, high-dose TXA was associated with a lower risk of bleeding compared to placebo (16.7% vs. 18.1%, relative risk, 0.92; 95% confidence interval, 0.81 to 1.05; *p* = 0.22), and a significantly lower total number of blood products used (4,331 vs. 7,994, *p* < 0.001), without an increased risk of death or thrombotic complications (22). However, TXA was associated with a higher risk of postoperative seizures. In patients undergoing cardiac surgery with cardiopulmonary bypass, high-dose TXA infusion compared with low-dose resulted in a statistically significant reduction in the proportion of patients transfused and was noninferior regarding a composite primary safety endpoint consisting of 30-day mortality, seizures, kidney dysfunction, and thrombotic events (23).

## Conclusion

**Figure 3:**
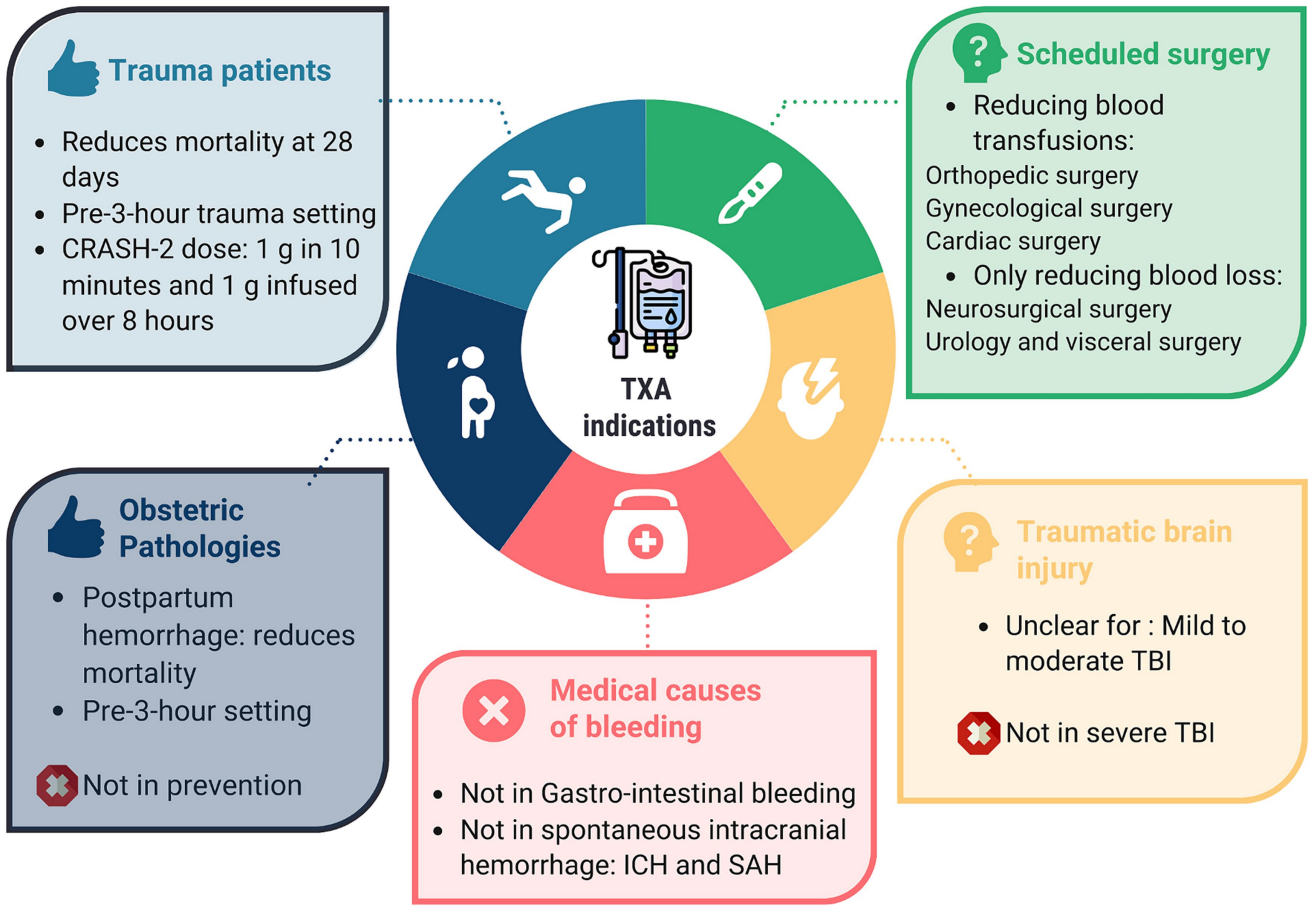
Summary of TXA’s main indications.

Tranexamic acid has been extensively evaluated in some of the largest trials in our field. Although the drug’s effect on strong outcomes may be modest in certain situations, its favorable safety profile allows it to be recommended for trauma patients, in obstetrics, and in scheduled surgeries at risk of bleeding. However, it cannot be recommended for spontaneous intracranial bleeding, subarachnoid hemorrhage (SAH), or gastrointestinal bleeding (Figure 3).

## Author contributions

MB: Writing – original draft, Writing – review & editing. AB: Writing – original draft, Writing – review & editing. PR: Writing – original draft, Writing – review & editing. YH: Writing – original draft, Writing – review & editing. AC: Writing – original draft, Writing – review & editing. AR: Writing – original draft, Writing – review & editing.
